# Mutagenesis of RpoE-like sigma factor genes in *Bdellovibrio* reveals differential control of *groEL* and two *groES* genes

**DOI:** 10.1186/1471-2180-12-99

**Published:** 2012-06-07

**Authors:** Carey Lambert, Rob Till, Laura Hobley, R Elizabeth Sockett

**Affiliations:** 1 Centre for Genetics and Genomics, School of Biology, University of Nottingham Medical School, QMC, Derby Road, Nottingham, NG7 2UH, UK; 2 College of Life Sciences, University of Dundee, Dow Street, Dundee, DD1 5EH, UK

## Abstract

**Background:**

*Bdellovibrio bacteriovorus* HD100 must regulate genes in response to a variety of environmental conditions as it enters, preys upon and leaves other bacteria, or grows axenically without prey. In addition to “housekeeping” sigma factors, its genome encodes several alternate sigma factors, including 2 Group IV-RpoE-like proteins, which may be involved in the complex regulation of its predatory lifestyle.

**Results:**

We find that one sigma factor gene, *bd3314,* cannot be deleted from *Bdellovibrio* in either predatory or prey-independent growth states, and is therefore possibly essential, likely being an alternate sigma 70. Deletion of one of two Group IV-like sigma factor genes, *bd0881*, affects flagellar gene regulation and results in less efficient predation, although not due to motility changes; deletion of the second, *bd0743*, showed that it normally represses chaperone gene expression and intriguingly we find an alternative *groES* gene is expressed at timepoints in the predatory cycle where intensive protein synthesis at *Bdellovibrio* septation*,* prior to prey lysis, will be occurring.

**Conclusions:**

We have taken the first step in understanding how alternate sigma factors regulate different processes in the predatory lifecycle of *Bdellovibrio* and discovered that alternate chaperones regulated by one of them are expressed at different stages of the lifecycle.

## Background

*Bdellovibrio bacteriovorus* HD100 must regulate genes in response to a variety of environmental conditions as it enters, digests, and leaves other Gram-negative bacteria, or when it grows axenically without prey [1-3]. Discrete waves of enzymes digesting different prey contents are required so that predatory enzymes do not act on each other, as the *Bdellovibrio* changes from a non-replicating “attack-phase” outside the prey, to a growing and replicating state inside prey. The *B. bacteriovorus* HD100 genome encodes several potential sigma factors for RNA polymerase which may contribute to such organised waves of gene regulation [4]. The *Bdellovibrio bacteriovorus* HD100 genome has several predicted “housekeeping” sigma factors: gene *bd0242* encoding an RpoD sigma 70 sigma factor; gene *bd3318*, encoding a FliA-like sigma factor and gene *bd0843* encoding an RpoN-like sigma factor. In addition, there are two homologues of genes predicted to encode Group IV-RpoE-like sigma factors, *bd0881* (product predicted at 162 amino-acids) and *bd0743* (product predicted at 206 amino-acids). Further, gene *bd3314* is predicted to encode a larger sigma factor homologue (predicted at 373 amino-acids) with sigma 70 homology.

RpoE-like sigma factors in other bacteria mediate gene expression in response to changes in host/external environment and bacteria with mutations in *rpoEs* can be defective in virulence or other host interactions [5]. Bd0881 and Bd0743 predicted proteins show significant homology (28.6% and 31.8% identity respectively) to the *rpoE* gene product of *E*. *coli* which encodes a sigma factor of the ECF type that is responsive to extra-cytoplasmic, periplasmic events; RpoE in *E. coli* is sequestered at the inner membrane by an RseA RseB pair of proteins, until inducing-events, in the shape of abnormally folded proteins in the periplasm, cause it to be released and active [6]. The *Bdellovibrio* genome, like that of other delta-proteobacteria, does not contain *rseAB* genes, suggesting that the RpoE-like sigma factors encoded by *bd0881* and *bd0743* belong more generally to the Group IV-type sigma factors. Unlike some members of this group, the *Bdellovibrio* genes lack the typical downstream co-transcribed gene encoding a product with homology to an anti-sigma factor. Indeed the genes (*bd0745* and *bd0882*) that are immediately downstream of *bd0743* and *bd0881* are unique to the *Bdellovibrio* genome, with no other significant homologues in other bacteria.

We hypothesised that the regulatory functions of alternate Group IV sigma factors might be diverse and important in the *Bdellovibrio* lifestyle, where prey-interaction versus prey-independent axenic growth brings with it many different challenges to the cell, including outer membrane insults, and a need for a great deal of *de novo* protein synthesis. Thus we used directed mutagenesis with kanamycin cartridge insertion, to test if inactivation of the three sigma factor genes *bd3314, bd0881* and *bd0743,* affected viability and to determine what their regulatory roles in the *Bdellovibrio* axenic and predatory lifestyles may be. We find that one is likely essential, one is involved in regulating predatory processes and one is involved in repression of different components of the GroESEL chaperone complex, which themselves may have different roles in the predatory lifecycle.

## Results

### Transcriptional studies and bioinformatics show the operon structures for *bd0743* and *bd0881*

**Figure 1:**
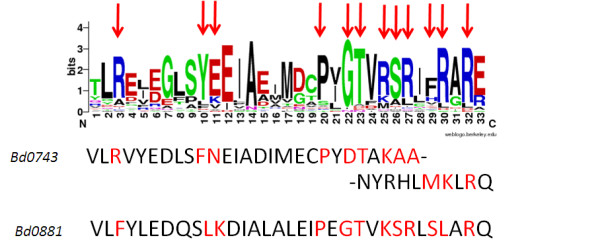
**Sequence LOGO showing DNA binding region of RpoEs **[8]**.** The first 35 sequences annotated as rpoE in the NCBI database were entered into the Weblogo program (http://weblogo.berkeley.edu/) using default parameters. The red arrows indicate the residues known to bind DNA in *E. coli*. The residues highlighted in red on the *Bdellovibrio* sequences show those that align to these using the ClustalW program and indicate that these are different from most RpoEs and each other, suggesting that they may well bind to different DNA motifs. There is also a 4 residue insertion in the Bd0743 sequence relative to the other sequences.

RT-PCR on total RNA taken from predatory growth conditions demonstrated that adjacent genes *bd0880bd0881* and *bd0882* are co-transcribed in an operon, but that *bd0743* was not co-transcribed with the adjacent gene *bd0745*. This differs to the situation for Group IV sigma factors in other bacteria where the downstream gene usually encodes an anti-sigma-factor [7]. Alignment of the RpoE protein from *E. coli* with the predicted gene products from *bd0743* and *bd0881* gave another indication that these *Bdellovibrio* proteins may have different roles from that of *E. coli* RpoE. Amino acids known to bind the −35 recognition site in *E. coli* differ in Bd0743 and Bd0881 as illustrated in Table 1 and Figure 1, suggesting that these sigma factors may recognise different sequences to those of *E. coli* and also to each other. Additionally *bd0881* is conserved in the genome of *Bacteriovorax marinus*, a marine *Bdellovibrio*-like bacterium but *bd0743* does not have a strong homologue in that genome. These data were provided by BLAST analysis hosted by the Wellcome Trust Sanger Institute and can be obtained from http://www.sanger.ac.uk/cgi-bin/blast/submitblast/b_marinus.

**Table 1:** **amino acid composition of −35 recognition sites of the*Bdellovibrio*sigma factor gene products compared to*E. coli*RpoE**[8]

**-35 recognition site amino acids in*E. coli RpoE***	**Corresponding amino acid in Bd0743**	**Corresponding amino acid in Bd0881**
**R149**	**R**	F
**Y156**	F*	L
**E157**	N	K
**P166**	**P**	**P**
**G168**	D	**G**
**T169**	**T**	**T**
**R171**	K*	K*
**S172**	A	**S**
**R173**	A	**R**
**F175**	M	S
**R176**	K*	L
**R178**	**R**	**R**

Many of the residues comprising the −35 recognition site of *E. coli* RpoE (bold) are not conserved in *B. bacteriovorus* HD100 (shown as non-bold), suggesting that these RpoE-like proteins may recognise different DNA consensus sequences and correlating with the lack of classical *E. coli* RpoE consensus sequences in promoters in the *B. bacteriovorus* HD100 genome. (* = conservative substitution)

### Inactivation of sigma factor genes suggests that *bd3314* may be essential

Kanamycin resistant cassettes were inserted into the *bd0743bd0881* and *bd3314* genes to disrupt their coding sequences, and knockout mutants were screened for as described previously [9]. Viable knock-out mutants capable of predation were obtained for *bd0743* and *bd0881*, but they could not be obtained for *bd3314*, despite extensive screening in axenic (prey-independent) and predatory conditions well beyond the bacterial numbers from which the other two mutants were isolated, suggesting that Bd3314 may be essential (a total of 287 isolates were screened from 4 separate conjugation experiments, yielding only *bd3314* merodiploids, compared to 10 and 29 isolates yielding 6 and 1 knock-out mutants for *bd0743* and *bd0881* respectively). Bd3314 is larger than the other RpoE-like sigma factors (predicted 373 amino acids compared to 162 and 206) with homology to regions 1.2, 2, 3 and 4 of sigma 70 and so this may be acting as an alternative sigma 70 factor guiding the transcription of housekeeping genes which would explain why generating a knock-out mutant was not obtained. Top hits from a BLAST search for Bd3314 are sigma-70 genes from many delta-proteobacteria, (outwith the predatory *Bdellovibrio*) further supporting its possible role as an alternative sigma 70 protein. Some hits from BLAST were annotated as RpoH, but Bd3314 is unlikely to be RpoH as it lacks the “RpoH box” conserved in these proteins [10]. Further studies on the groups of genes it regulates is beyond the scope of this manuscript, but it is likely that as Bd3314 is conserved in other delta-proteobacteria, including many non-predatory bacteria, it may not have a specialised predatorily associated function.

### Luminescent prey assay shows less efficient predation by a *Bdellovibrio bd0881* knockout strain

**Figure 2:**
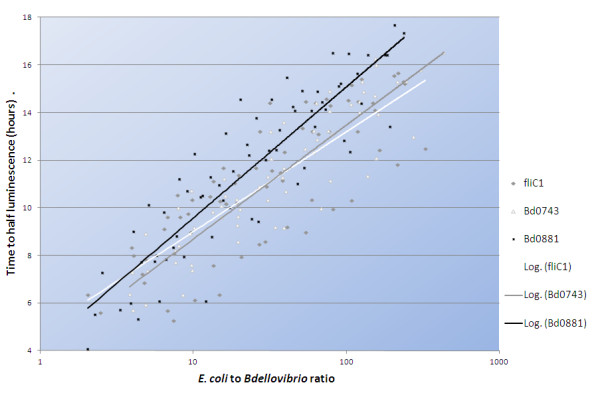
**Predation efficiency assay using luminescent prey shows reduced efficiency for the** Δ**Bd0881 mutant.** Predatory efficiency plot showing log_10_ initial ratios of prey to predator against time to reach half of starting luminescence for the strains. Equivalent numbers of the ΔBd0881 mutant *Bdellovibrio* killed the prey cells more slowly than ΔBd0743 or kanamycin resistant “reconstituted wild-type”, fliC1 merodiploid strain.

Both the ΔBd0743 and ΔBd0881 knockout strains were able to grow predatorily but a predation efficiency assay [9] using luminescent prey cells showed that the ΔBd0881 mutant was less efficient at predation upon *E. coli* than the ΔBd0743 mutant and the wild-type control (Figure 2). For any given ratio of *E. coli* to *Bdellovibrio,* the ΔBd0881 strain took longer to reduce light emitted from the luminescent *E. coli* to half of its maximum, and hence took longer to kill the prey. An extra sum of squares F test carried out using the GraphPad Prism 5 software showed that this difference was significant (P < 0.0001). This suggests that Bd0881 controls, or optimises, the transcription of some genes involved in the predatory lifestyle while Bd0743 does not and thus Bd0881 is the first experimentally identified *Bdellovibrio* transcriptional regulator of predation genes. Axenic, prey-independent growth of both mutants was not significantly different from wild-type and heat shock (at 42°C for 10 min) did not reduce viability suggesting that they are not acting as typical alternate sigma32-like factors.

**Figure 3:**
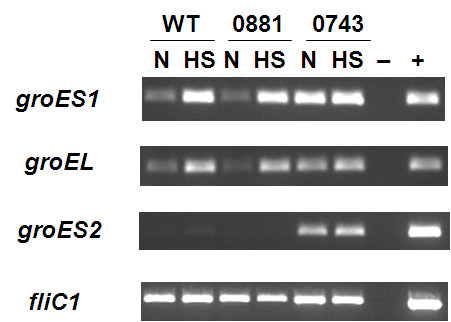
**RT-PCR showing relative levels of transcription of chaperonin and flagellin genes in total RNA from attack phase*Bdellovibrio*, under normal and heat-shocked conditions.** RT-PCR with transcript specific primers was carried out on matched concentrations of RNA (matched by Nanodrop spectrophotometer readings) from wild-type and mutant attack-phase *Bdellovibrio* including samples subjected to heat shock (42°C for 10 minutes). Total RNA samples from :-WT- wild-type HD100 attack phase, N- non-heat shocked 29°C, HS- heat shocked at 42°C for 10 minutes, 0881- ΔBd0881 attack phase, 0743- ΔBd0743 attack phase, Lane 7- no template negative control, Lane 8- HD100 genomic DNA positive control. “No reverse transcriptase” controls were performed for each template and were negative for DNA contamination (data not shown). The abundant transcript produced using primers designed to anneal to the *fliC1* gene acts as a positive control by showing that there was ample total RNA in all samples.

Previously we have shown [11] that predatory efficiency in liquid media can be affected by the swimming speed of the *Bdellovibrio* which affects how efficiently they enter areas where they collide with prey. Interestingly, the transcription of the *bd3052 fliC5* flagellin gene was found, by RT-PCR on attack phase *Bdellovibrio* RNA, (Figure 3) to be significantly down regulated in the ΔBd0881 mutant compared to the ΔBd0743 mutant and the wild type (WT) HD100 under heat shock conditions. This suggests that Bd0881 may have some role in regulating the expression of *fliC5*, altering protein composition and thus rigidity and/or the lengths of flagellar filaments in *Bdellovibrio*.

**Figure 4:**
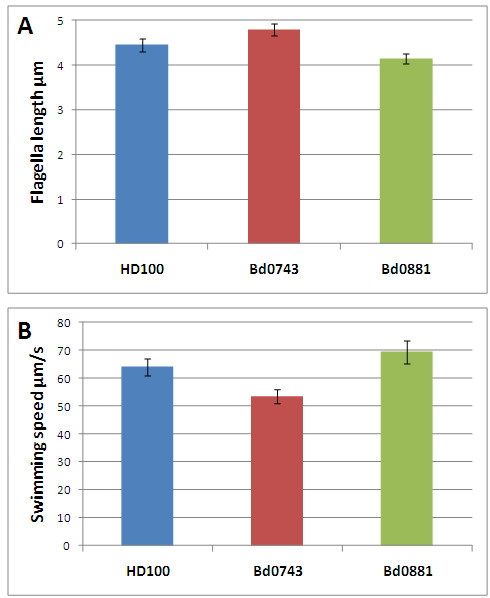
**Lengths of flagella and swimming speeds of the mutants and wild-type. A-** Flagellar length of wild type and sigma factor mutants measured from electron micrographs, error bars show 95% confidence intervals. **B-** Speeds of wild type and mutant predatory strains measured by the Hobson Bactracker, error bars show 95% confidence intervals.

A comparison of the flagellar lengths of the two strains versus WT, at the exact same growth conditions, revealed that the flagellar filaments of ΔBd0881 were slightly but significantly (P = 0.0026), shorter than those in wild type *Bdellovibrio*. In contrast, those in ΔBd0743 were longer (P = 0.0016) than the wild type (Figure 4A). We have previously shown [11] that *fliC5* deletion shortens flagella and that Δ*fliC5* flagellar mutants swim more slowly and prey less efficiently on *E. coli* in the luminescent prey assay. Interestingly, when we compared the swimming speeds of the two strains (Figure 4B) we found that the ΔBd0881 cells swam significantly (P = 0.044) but only slightly faster than the wild type, however, surprisingly both swam significantly (P < 10^-5^) faster than the ΔBd0743 strain despite it having longer flagellar filaments. Thus having a changed flagellin composition in the ΔBd0743 mutant strains produced a longer flagellum but either it had a “flaccid” wave form structure that produced less torque and thus swimming speed, or the ΔBd0743 mutation affected its complement of motor proteins so that the longer flagellum in this strain rotated slower than the wild type. We couldn’t test this by antibody-tethering cells by their flagella to glass slides because the flagella are sheathed with an outer membrane. The effect was a subtle one as the speeds were only slightly altered and the flagella waveforms did not appear to be grossly altered by electron microscopy.

To look for any evidence of association between RpoE-like sigma factor proteins and motility gene expression, we firstly measured the transcription of the 3 *motA* genes in ΔBd0881 and ΔBd0743, but found no difference compared to wild type (data not shown). This led us to conclude that Bd0881 does not act at motor regulation and does not produce faster rotating but shorter flagella.

**Figure 5:**
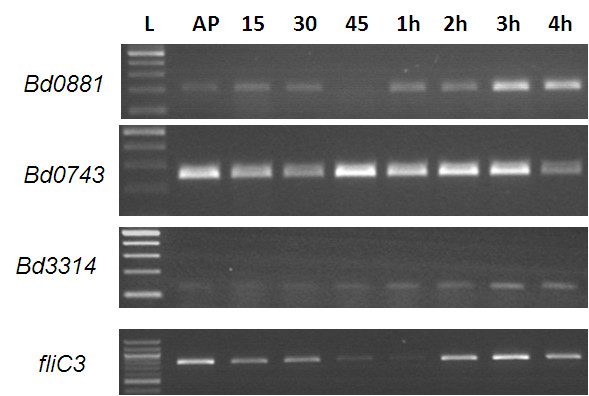
**Expression patterns of*rpoE*-like genes compared to*fliC3*in total RNA taken from across the predatory cycle studied by RT-PCR.** RT-PCR with transcript-specific primers on total RNA prepared from identical numbers of *B. bacteriovorus* HD100 predator synchronously invading an *E. coli* S17-1 prey culture, with samples taken as the predatory infection, and *Bdellovibrio* development proceeds across a time course. L- NEB 100 bp ladder, AP- attack-phase 15–15 minutes predation, 30–30 minutes predation, 45–45 minutes predation 1-4 h: 1,2,3,4 hours predation respectively. Controls of no template, no reverse transcriptase, *E. coli* S17-1 only RNA as template and *B. bacteriovorus* HD100 genomic DNA were carried out. Primers designed to *bd0743* give a product in every sample, thus act as a positive control for the RNA, validating the lack of expression in some of the samples. A similar expression pattern was seen for *bd0881* and *fliC3*.

We next tested whether there was an association between the transcriptional expression profiles of the *rpoE-like* genes and flagellar genes, measuring this by RT-PCR in total RNA from across the predatory cycle (Figure 5). We found that the expression patterns for *bd0743* and *bd3314* were constitutive but the expression pattern of *bd0881* was similar to that seen for the key *fliC*3 gene of *Bdellovibrio*[11]; *fliC3* is the only flagellin gene (from 6 *fliCs*) whose expression is crucial to flagellar synthesis, and its repression prevents motility of *Bdellovibrio*[6].

Our results showed that expression of *bd0881* was all but abolished at 45 min to 1 hour after *Bdellovibrio* addition to prey, and resumed later in the predatory cycle, before prey lysis, as shown in Figure 4 alongside expression of the critical *fliC3* gene. The expression of the *fliC3* gene initially drops early in the predatory cycle, then resumes as the *Bdellovibrio* are nearing septation and flagella are synthesised prior to prey lysis and progeny escape from the prey cell debris into liquid cultures. Thus the similarity in expression patterns of *fliC3* gene and *bd0881* during predation may imply that Bd0881 protein is involved in regulatory events to do with the timepoints where flagella are being synthesised, i.e. around septation, but the fact that ΔBd0881 mutants are not immotile shows that Bd0881 is not required for the “all or nothing” induction of the *fliC3* gene expression itself.

### RT-PCR reveals regulation of chaperone genes by Bd0743

RT-PCR was used to study the expression of GroE chaperone protein genes in wild-type and sigma-factor knockout *Bdellovibrio* strains, as chaperone genes are typically RpoE-regulated in other bacteria, although no obvious *E. coli* RpoE- like consensus sequence was seen upstream of them in the *B. bacteriovorus* HD100 genome. Other bacteria induce expression of GroE protein chaperones upon heat shock (typically experimentally 42°C) in order to deal with misfolded proteins [12]. Furthermore, over-expression of chaperones can aid the expression of high levels of proteins in cells [13] including situations where addition of phage–encoded GroES proteins modify the size of protein that the bacterial chaperone can fold, to assemble large phage capsid proteins [14]. The *Bdellovibrio* genome has, in addition to the *bd0097bd0099 groES groEL* genes, a second homologue, *bd3349*, of *groES* (here designated *groES2* versus *groES1* for *bd0097*), so we investigated the expression of all these genes by RT-PCR using matched amounts of RNA from wild-type and sigma-factor mutant *Bdellovibrio*, treated in attack phase, at different temperatures (29°C and heat-shock 42°C for 10 mins; Figure 3) using methods previously described [15]. In wild-type *Bdellovibrio*, as is the case in many other bacteria, *groES1EL* expression was low at normal *Bdellovibrio* growth temperature (29°C) and expression was induced at a higher level under heat shock (42°C). This situation was the same for wild type and the ΔBd0881 mutant indicating that the Bd0881 sigma factor is not involved in this heat shock event. In the ΔBd0743 mutant, however, *groES1EL* expression was de-repressed, even in non-heat shock conditions suggesting that the Bd0743 sigma factor controls, directly or indirectly, the repression of *groES1EL* under normal temperature conditions. The viability of the ΔBd0743 cells was not affected under predatory growth conditions as determined by plaque assay indicating that this GroE deregulation does not severely affect the cells during laboratory culturing.

The second chaperone gene *groES2* (*bd3349*) was expressed at a very low level, in attack phase cells of in the wild-type and ΔBd0881 mutant, under both normal and heat shock conditions,(Figure 3); suggesting that possibly it is not normally part of the heat shock response and may have a different role outside. In the ΔBd0743 mutant, however, *groES2* expression was de-repressed in both normal and heat shock conditions, again implying that this sigma factor controls the expression of repressors of chaperone gene expression.

**Figure 6:**
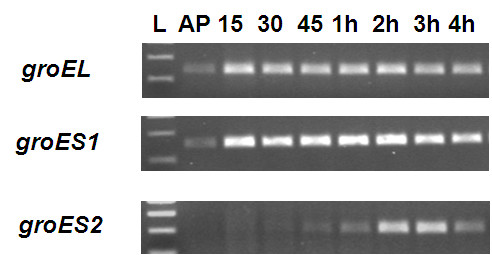
**Transcriptional expression patterns of the three*Bdellovibrio*chaperonin genes across the wild type predatory cycle.** RT-PCR with transcript-specific primers was performed on total RNA prepared from identical volumes of *B. bacteriovorus* HD100 predator with *E. coli* S17-1 prey infection culture as the predatory infection proceeds across a time course. L- NEB 100 bp ladder, AP- attack-phase 15–15 minutes predation, 30–30 minutes predation, 45–45 minutes predation 1-4 h: 1,2,3,4 hours predation respectively. Controls of no template, no reverse transcriptase, *E. coli* S17-1 only RNA as template and *bacteriovorus* HD100 genomic DNA were carried out. Primers designed to *bd0097 groES1* and *bd0099 groEL* give a product in every sample, thus act as a positive control for the RNA, validating the lack of expression of *bd3349 groES2* in the earlier part of the infectious cycle.

**Figure 7:**
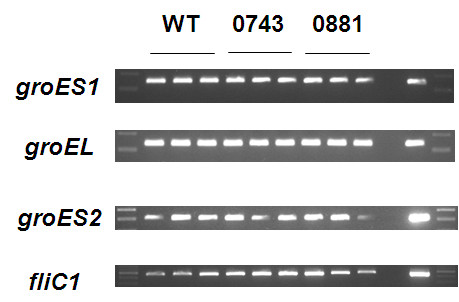
**Transcriptional expression patterns of the three*Bdellovibrio*chaperonin genes during axenic Host-Independent growth.** RT-PCR with transcript specific primers was carried out on matched concentrations of RNA (matched by Nanodrop spectrophotometer readings) from axenically grown Host-Independent *Bdellovibrio*. Three independently isolated strains of each sigma factor mutant and each host-independent (HI) wild-type were used to account for HI strain-to strain variation. L- NEB 100 bp ladder –ve - no template negative control + ve- HD100 genomic DNA positive control.

Recent work [16] has demonstrated different roles for multiple chaperone *groEL* gene products in the predatory *Myxococcus* including differential roles in predation and so it is possible that a similar situation exists with the duplicate *groES* gene products of *Bdellovibrio*. The *groES2* gene was annotated in the *B. bacteriovorus* HD100 genome as encoding a 224 amino acid protein, but closer inspection reveals that a more likely start codon is at the methionine at base pair position 322 within this orf as the region before this, in the old annotation, includes lots of repetitive sequence. Using this start codon, the predicted protein of 117 amino acids has 34% identity and 62% similarity with the predicted (100 amino-acid) GroES protein of *E. coli,* and this 117aa region only of Bd3349 GroES2 is homologous to all predicted GroES sequences of delta-proteobacteria which give the highest BLAST homology scores for the Bd3349 protein. RT-PCR primers for *groES2* were designed to anneal to RNA encoding this orf and transcription of both *groES* genes was monitored in RNA extracted over a wild type predatory time-course of *B. bacteriovorus* HD100 preying upon *E. coli* (Figure 6). This showed that *groES1* was upregulated early at 15 minutes upon *Bdellovibrio* contact with prey cells and when the *Bdellovibrio* were growing within prey, remaining constitutively expressed throughout the predatory cycle. In contrast *groES2* was not expressed early but was upregulated later, at 2–4 hours in the predation cycle when *Bdellovibrio* were beginning to septate and lyse prey. Although there are more *Bdellovibrio* present at this stage of the predatory cycle as a result of replication within the prey, the upregulation is unlikely to solely be a result of this as *groES2* is not expressed at all in earlier stages of the cycle and so its induction here is significant. RT-PCR was also performed on matched amounts of RNA derived from 3 different host-independent strains derived from each sigma-factor mutant and a control wild-type (Figure 7) and revealed that *groEL, groES1* and *groES2* were all expressed at similar levels in each of the mutants in axenic, prey-independent (HI) growth. As (HI) host-independently growing *Bdellovibrio* populations include a mixture of attack phase and filamentous growth stage cells, it is not surprising that all of the chaperones are expressed in these cells.

## Conclusions

We have shown that of three *B. bacteriovorus* HD100 sigma factor genes with at least partial *rpoE* homology, one- *bd3314*, is likely essential for *Bdellovibrio* cell life and cannot be deleted. *bd0881* and *bd0743* can be deleted with the *Bdellovibrio* retaining the ability to grow predatorily or prey-independently.

In the case of ΔBd0881 the predatory efficiency was reduced, despite the flagellar motility of the mutant being slightly increased, (despite a slight but statistically significant shortening of flagellar filament length) thus the change in predation efficiency may not be due to motility changes but regulation of other predatory genes. The *bd0881* gene has an expression pattern across the predatory cycle that is similar to that of the flagellin genes whose expression is required for *Bdellovibrio* motility*.* That *bd0881* expression is turned off and then resumes at a similar time to flagellin gene expression, during the predatory cycle, implies that Bd0881 may have a role associated with pre-septation developmental maturation of *Bdellovibrio* around the time that flagella are being built in newly dividing cells. However the Bd0881 sigma factor does not directly regulate the expression of *fliC* flagellin or *mot* flagellar motor genes themselves.

Surprisingly, predatory efficiency was not affected in our cultures by the slower swimming speed of the ΔBd0743 sigma factor mutant; this is probably indicative of sufficient mixing of predator and prey at close quarters in lab conditions. The slight increase in flagellar length in ΔBd0743 mutants is likely to have come with the incorporation of a higher percentage of a less rigid flagellin in the flagella causing a less efficient “bow wave” and this may account for the slower swimming. In both the ΔBd0743 and ΔBd0881 mutants, small but significant changes in swimming speed were paradoxically associated with changes *apparently in the wrong direction* in flagellar length. This shows that it is not simply flagellar length that governs the thrust produced by flagellar propellers. In previous studies on the six different flagellins that are incorporated into the flagellar propeller of *Bdellovibrio*[11,17], we found that different flagellin compositions of a single *Bdellovibrio* flagellum are possible, and that in the case of a *fliC4* mutant, for example, an apparently wild type-length flagellum gave a lower swimming speed than wild type [11] suggesting an altered filament rigidity. As flagellar filament growth, in a bacterium with six flagellins, is a post-transcriptionally highly controlled process involving diverse chaperones and gate keepers at the base of the flagellum allowing different subunits to be added into the growing flagellum [18] we cannot expect to tell anything meaningful about these small changes of swimming speed from simple studies of flagellar filament gene expression, so we have decided to leave this aspect of the investigation at this point.

In looking at chaperonin expression regulation by *B. bacteriovorus* HD100 sigma factors, we found that, in contrast to *bd0881*, deletion of which had no effect, the product of gene *bd0743* acts more like the heat shock sigma factor RpoE of other bacteria and represses (directly or indirectly) the level of expression of chaperonin genes *groES1 groEL* (*bd0097* and *bd0099*) in non-heat shock conditions and the level of expression of the *groES2* (*bd3349*) gene under both heat-shock and non-heat-shock conditions. These data and the finding that the *groES2* gene is normally expressed in wild type *Bdellovibrio* only during the late stages of predation (2–4 hours) when the *Bdellovibrio* are septating and preparing to lyse the exhausted prey bdelloplast, may suggest that a modified chaperonin complex involving GroES2 is used in *Bdellovibrio* protein expression and folding that occurs at this point. Ascertaining why this is the case requires more chaperone-specific experimentation, beyond the scope of this study and mutagenesis of *bd3349* is underway. That the majority of GroES residues shown to interact with GroEL in *E. coli*[19] are conserved or have conserved substitutions in both of the GroES1 and GroES2 homologues of *B. bacteriovorus* HD100 supports the idea that they form genuine alternative chaperonin complexes, making GroEL protein folding chambers with different GroES “lids”. It is a tantalising possibility that *Bdellovibrio* has a requirement for a modified chaperonin complex for the folding of unusual *Bdellovibrio* proteins required for late-stage prey lysis or *Bdellovibrio* attack phase cell maturation. The Bd0743-controlled, late-stage expression of *groES2* is a possible mechanism for this. Although the (reannotated) *Bdellovibrio groES2* gene product is larger at 117 amino-acids than the *bd0097 groES1* gene product which is 100 amino-acids, there is no significant additional homology (above that for GroES1) between *Bdellovibrio* GroES2 and the bacteriophage T4 Gp31 GroES-like protein (data not shown). The bacteriophage T4 Gp31 GroES-like protein allows formation of a larger protein folding chamber for unusual phage capsid protein Gp23 to fold. *Bdellovibrio*, being a bacterium rather than a phage, does not have any homologues of this protein, so any analogous alternative role for GroES2 in *Bdellovibrio* protein folding awaits the outcomes of further mutagenesis studies.

## Methods

### Strains and growth conditions

A list of bacterial strains used in this study is presented in Table 2. *E. coli* was grown on YT media overnight (about 16 hours) with 50 μg ml^-1^ kanamycin sulphate as appropriate. Host dependent, predatory *Bdellovibrio* were grown in liquid prey lysate cultures in Ca/HEPES buffer or on YPSC double agar overlays as described elsewhere [20].

**Table 2:** List of strains used in this study

**Strain**	**Description**	**Reference**
*E. coli* S17-1	*thi,pro,hsdR*^-^,*hsdM*^+^,*rec*A; integrated plasmid RP4-Tc::Mu-Kn::Tn*7*	[21]
*E. coli* DH5α	F’ *endA1 hsdR17* (r_k_^-^m_k_^-^) *supE44 thi-1 recA1 gyrA* (Nal^r^) *relA1* Δ(*lacIZYA-argF*) U169 *deoR* (ϕ80d*lac*Δ(*lacZ*)M15)	[22]
*E. coli* S17-1: pZMR100	Plasmid vector used to confer Km^r^ on S17-1 & DFB225 that are being used as prey for Km^r^*Bdellovibrio strains*	[23]
*Bdellovibrio bacteriovorus* HD100	Wild-type	[4]
*Bdellovibrio bacteriovorus fliC1* merodiploid	Km^r^ derivative of HD100 merodiploid for *fliC1*	[24]
*Bdellovibrio bacteriovorus bd0743*	HD100 *bd0743::aphII*	This study
*Bdellovibrio bacteriovorus bd0881*	HD100 *bd0881::aphII*	This study

### RNA isolation and RT-PCR

Total RNA was isolated with modifications of the Promega SV total isolation kit described previously [11]. Heat shock was carried out by incubating 20 ml of prey-dependent *Bdellovibrio* in 50 ml centrifuge tubes at 29°C, then transferring to a 42°C water bath (with a control transferred to a 29°C water bath) for 10 minutes before adding 5 ml 5% phenol 95% ethanol (v/v) and proceeding with RNA extraction. Plaque enumeration confirmed that this heat treatment had no significant affect on cell viability. RT-PCR was carried out with the Qiagen one-step RT-PCR kit according to the manufacturer’s instructions as described elsewhere [25]. Primers used are shown in Table 3.

**Table 3:** List of primers used in this study

**Primer**	**Sequence**	**Use**
fliC3RTF	ATGCTCAGAGAGTTCTCTGG	*fliC3* RT-PCR
fliC3RTR	AATGACTTGTTCAAGAGTCC	*fliC3* RT-PCR
fliC5RTF	GCTCAACGTAACTTGGTCGG	*fliC5* RT-PCR
fliC5RTR	AGCCGATCAGCTTAAGAGCC	*fliC5* RT-PCR
bd0881RTF	CGCAAGGAAGAAGTCAGTCC	*bd0881* RT-PCR
bd0881RTR	CAGGCTTAAACGGGATTTCA	*bd0881* RT-PCR
bd0743RTF	GCTCTTTTTCCGAACTCGTG	*bd0743* RT-PCR
bd0743RTR	TACAGCCAATTGCACATCGT	*bd0743* RT-PCR
Bd3314RTF	GGATTCGCGGCTATATTCAA	*bd3314* RT-PCR
Bd3314RTR	TGGCATCCAGAGCTTCTTTT	*bd3314* RT-PCR
fliC1RTF	GCATCTATCGCAGCACAACG	*fliC1* RT-PCR
fliC1RTR	CCGTCGAGTCGGCATCAAAT	*fliC1* RT-PCR
Bd743-F	GAAATTCTTGAAGCCATGACCAATGCG	Cloning *bd0743*
Bd743-R	CGGGATCCGAGTGGCCTCTGGATTCG	Cloning *bd0743*
Bd881-F2	CGGAATTCTGGTCGCAAGAATATCTGCC	Cloning *bd0881*
Bd881-R2	GCTCTAGAATGACTCCAAGCTGGTTGGC	Cloning *bd0881*
Bd3314-F	GCTCTAGACAGAAAGGAAACGACGCAC	Cloning *bd3314*
Bd3314-R	GCTCTAGAGCTTAGGGGTTCTGTATAA	Cloning *bd3314*

### Gene knock-out and luminescent prey assay

Kanamycin resistance cassettes were inserted into the *rpoE-*like sigma factor genes of *Bdellovibrio*, as described elsewhere [9,11]. Primers used are listed in Table 3. Luminescent prey assays (with *E. coli* S17-1 containing the plasmid pCL100) were carried out as described elsewhere [9,11] except using a Fluostar Optima machine and the final enumeration data were expressed as *Bdellovibrio* per *E. coli*. An extra sum of squares F test carried out using the GraphPad Prism 5 software was carried out to show significance.

### Electron microscopy and flagella filament length analysis

*Bdellovibrio* cells were incubated for 24 hours in a predatory culture before being placed on a carbon formvar grid (Agar Scientific), and stained with 0.5% uranyl acetate pH 4.0 as described previously [17]. Cells were imaged using a JEOL JEM1010 transmission electron microscope. Flagellar lengths were measured to the nearest 0.01 μm for an average of 50 cells per strain, error bars show the 95% CI around the mean for each sample as described previously [17]. Student’s *t*-test was carried out to determine significance of results.

### Hobson BacTracker analysis of *bdellovibrio* swimming speeds

The swimming speed of each *Bdellovibrio* strain was analysed using Hobson BacTracker (Hobson Tracking Systems, Sheffield, United Kingdom) exactly as described in [24], including the use of the lower run speed limit of 15 μm/s to reduce the influence of Brownian motion, and accidental tethered-cell-body rotation, on the speed outputs. Cells were pre-grown for 24 hours in a typical 10 ml predatory culture with *E. coli* S17-1 as prey under the same conditions as for the electron microscopic analysis above. Student’s *t*-test was carried out to determine significance of results.

## Abbreviations

Rpo, RNA polymerase; E. coli, Escherichia coli; ECF, Extracytoplasmic Function; RT-PCR, Reverse Transcriptase Polymerase Chain Reaction; BLAST, Basic Local Alignment Search Tool.

## Competing interests

The authors declare that they have no competing interests.

## Authors contributions

RES designed the experiments and co-authored the manuscript. CL performed the RT-PCR and luminescence assays and co-authored the manuscript, RT constructed the mutants and performed RT-PCR, LH performed the electron microscopy and speed measurements. All authors read and approved the final manuscript
